# Evaluating the indigenous response to COVID-19 in rural Oaxaca, Mexico

**DOI:** 10.7189/jogh.13.03051

**Published:** 2023-10-06

**Authors:** Jeffrey H Cohen, Andrew P Mitchel, Francisco A Montiel Ishino

**Affiliations:** 1 Department of Anthropology, The Ohio State University, Columbus, Ohio, USA; 2 Department of Anthropology, The Ohio State University, Columbus, Ohio, USA; 3 National Institute of Environmental Health Sciences, Epidemiology Branch, Division of Intramural Research, National Institutes of Health, RTP, North Carolina, USA

**Figure:**
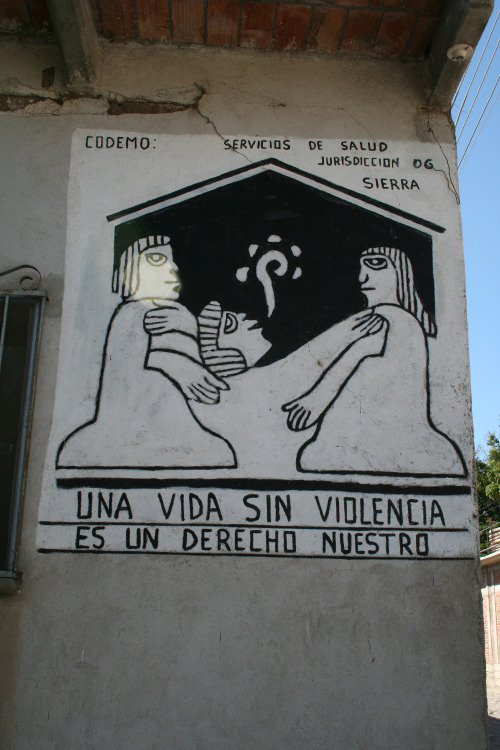
Photo: A public health billboard, Oaxaca, Mexico. Source: Photograph by JH Cohen.

The novel coronavirus 2019 (COVID-19) pandemic caused by the severe acute respiratory syndrome coronavirus 2 (SARS-CoV-2) has faded from the headlines as infection and mortality rates decline, yet remains a challenge to rural and indigenous communities in locations like Oaxaca, Mexico [1].

Rurality and indigeneity create further obstacles to communities’ effective response to the continued impacts of the pandemic [2]. Deep-seated social inequalities that are founded in rurality and ethnic heritage are exacerbated by comorbidities, limited access to health care, and health education [3,4], as well as poor sanitation and the lack of potable water. We review three continuing challenges that the COVID-19 pandemic poses for rural, indigenous communities in Oaxaca, Mexico.

First, we explore the direct, tangible impacts of the pandemic that rural, indigenous communities in Oaxaca endured from the multiple waves of COVID-19. The initial response of rural villages to COVID-19 was to support masking and social distancing, while simultaneously closing community access points with blockades and manned checkpoints. Consequently, rural indigenous communities throughout Oaxaca were largely unaffected in the early days of the pandemic. According to the Consejo Nacional de Ciencia y Tecnología (CONACYT), Mexico’s premier entity for the promotion of scientific and technological advances, and its tracking site [5], official rates of infection through mid-May 2020 remained low, especially in Oaxaca, with most indigenous communities registering no or very few cases until July 2020.

Despite proper infection prevention protocols, the rapid upsurge in the infection rate and virulence from the second and third waves of the pandemic [1] created a negative synergism for increased morbidity and mortality, partially due to inadequate governmental public health policies and education. The infection rates more than doubled from fall 2020 to winter 2021. We followed the trends in Oaxaca City, the state’s capital, to illustrate the change: We identified only 11 cases in April 2020, yet this number increased to 12 873 cases by April 2021 and further by about 250% through April 2022, reaching 32 411 cases. By April 2022, the mortality rate for indigenous Mexicans was 68% higher than for non-indigenous Mexicans [6].

Rapid growth in infections and mortality occurred outside of the city as well. While infection and mortality cases in rural Oaxaca appeared small, as most communities include populations in the hundreds to a few thousand, the impacts were no less challenging. The surge in virulence and infections was compounded by other health concerns and comorbidities (eg, type 2 diabetes mellitus) [7]. Poverty, ever a critical issue, limited access to vaccines and, together with limited access to affordable food and work [8], further increased the population’s vulnerability. Yet despite these hardships, the pandemic offered an opportunity for the Mexican Federal and Oaxacan State governments to reconsider the meaning of equal access to health care and equity [9]. It remains unclear if the policies and promises made by governmental agencies and NGOs were kept and if the planned programmes reached their target audiences or if they are still ongoing.

The pandemic’s direct and tangible impacts on indigenous Oaxacan communities are well captured in the numbers of infected cases and mortality rates [1]. The lack of adequate and accessible health care, as well as limited infrastructure and transportation, contribute to an increasing array of indirect challenges that negatively affect access to markets, including the tourism market which is critical for the economic well-being of many rural Oaxacans.

Indirect impacts include secondary events and outcomes that are not directly caused by the pandemic or are not tangible. Market women who depend on a steady stream of clientele noted that the pandemic forced them to rebalance work against limits that locked down marketplaces [10]. Indirect effects can be less obvious, as they are founded in historical patterns of inequality [11]. Indigenous and rural Oaxacans approach Federal and State programs with mistrust following generations of abuse, which limits the adoption of new solutions to health challenges and new technologies critical to well-being. The scepticism of rural Oaxacans to federal/external programmes is not new [12] and is manifest in poverty, poor infrastructure, as well as a system that is mired in 1940s corporatist politics [13-15].

Indirect forces complicated local pandemic responses as indigenous communities focused on the tangible, COVID-19-related challenge. This was clear during the first pandemic wave, as local actions played out with little Federal and State engagement. Unanticipated challenges continued to emerge as the new COVID-19 waves threatened rural communities and stressed already limited support by Federal and State programs [16].

In 2021, we documented the community response of rural Oaxacans to COVID-19 and noted that limiting the movement of people and building upon traditional forms of cooperation was critical to managing the pandemic [17]. While these actions slowed infection rates, cutting off access to communities made it hard to shop, reach schools and jobs, and stay in contact with family members who had relocated. The isolation interrupted access to markets, schools, and public transportation as bus networks, private cabs, and *collectivos* (taxis that carry multiple passengers) closed or functioned on reduced schedules. Work that would take villagers outside of their communities disappeared, as did jobs in the city due to the steep decline in tourism [18]. Limited access to health care, mental health care and educational programming, which were poor before the pandemic, did not improve and have not rebounded.

The COVID-19 pandemic overwhelmed the capacities of most communities, taxed limited resources, and changed how rural and indigenous Oaxacans think about their place in the world. Indigenous Oaxacans responded to the pandemic quickly and effectively. The actions of most communities mitigated the early impacts of the coronavirus through the first wave of the pandemic. Viral mutations and subsequent infection waves meant that rural communities were forced to respond anew with each new iteration of the virus. For many communities, these subsequent waves have proved devastating.

Waning interest in the pandemic and in support of related research complicates the development of a just, inclusive, and non-discriminatory framework. A more thoughtful, reasonable, and equitable intervention in a space like rural, indigenous Oaxaca would be to incorporate local voices, as well as formulate policies that account for local needs and consider local ways of dealing with illness and disease, which proved effective in the past.

Indigenous people have struggled with a history of exclusion, social injustice, inadequate health care, as well as limited access to services and technology. Local traditions hold some promise. Oaxacans at home and abroad depend on family and community-based reciprocity to organize amongst themselves. These connections work, whether they are found in a small rural community in Oaxaca or as part of a neighbourhood in downtown Los Angeles, California. To prepare for the next pandemic, we must build upon the experience of these communities to develop public health programming and opportunities in anticipation of a more effective response.
